# Primary bilateral adrenal B-cell lymphoma associated with EBV and JCV infection

**DOI:** 10.1186/1750-9378-4-1

**Published:** 2009-01-15

**Authors:** Luisa Barzon, Marta Trevisan, Filippo Marino, Vincenza Guzzardo, Giorgio Palù

**Affiliations:** 1 Department of Histology, Microbiology and Medical Biotechnologies, University of Padova, I-35121 Padova, Italy; 2 Department of Pathology, University of Padova, I-35121 Padova, Italy

## Abstract

Primary lymphoma of the adrenal gland is a rare and highly aggressive disease, with only a few reports in the literature. The pathogenesis is unknown, but detection of Epstein Barr virus (EBV) genome sequences and gene expression in some cases of primary adrenal lymphomas suggested the virus might be a causative agent of the malignancy. While investigating the presence of genome sequences of oncogenic viruses in a large series of adrenal tumors, both EBV and JC polyomavirus (JCV) DNA sequences were detected in a diffuse large primary bilateral B-cell non-Hodgkin lymphoma of the adrenal gland, which was diagnosed only at postmortem examination in a 77 year-old woman with incidentally discovered adrenal masses and primary adrenal insufficiency. The presence of both EBV and JCV genome sequences suggests the relevance of EBV and JCV coinfection in the pathogenesis of this rare form of B-cell lymphoma.

## Findings

Primary lymphoma of the adrenal gland is a rare and highly aggressive disease, with only a few reports in the literature [1-3]. It generally affects elderly individuals, especially those with a history of cancer, HIV infection, or autoimmune disorders, and, in about 50% of cases, it presents with symptoms of adrenal insufficiency due to bilateral adrenal involvement. Histologically, the most common type of primary adrenal lymphomas is diffuse large B-cell lymphoma [3]. Treatment with chemotherapy according to the CHOP-regimen (cyclophosphamide, doxorubicin, vincristine, prednisone) alone or in combination with rituximab has led complete or partial remission is some patients, even though prognosis remains poor with death occurring within one year after diagnosis [3-5]. The pathogenesis is unknown, but detection of Epstein Barr virus (EBV) genome sequences and gene expression in 9 out of 20 cases of primary adrenal lymphomas suggested the virus might be the causative agent of the malignancy [6]. While investigating the presence of genome sequences of oncogenic viruses in a large series of adrenal tumors and their potential association with malignancy [7], both EBV and JC polyomavirus (JCV) DNA sequences were detected in the case of primary bilateral adrenal B-cell lymphoma we are reporting here.

**Figure 1:**
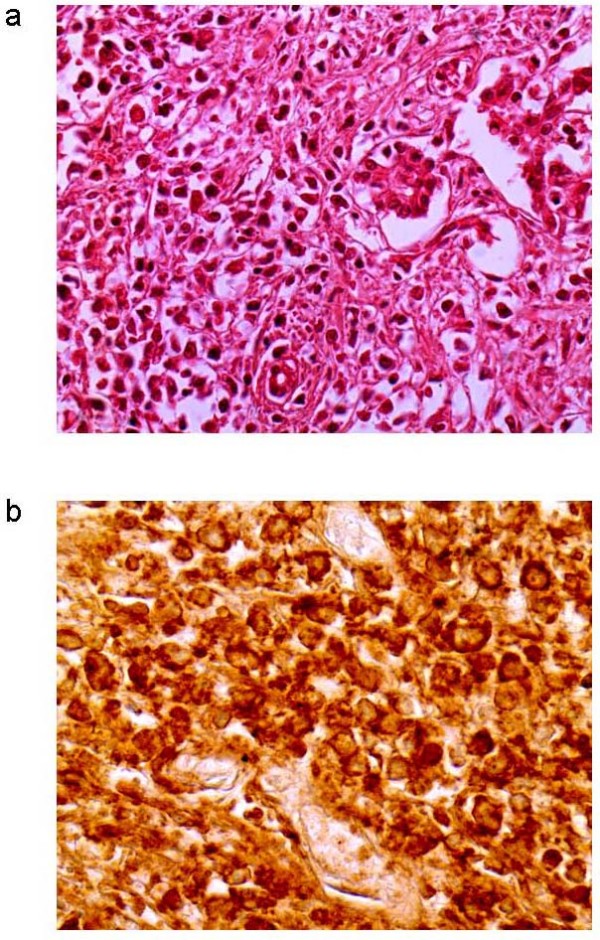
**a) Non-Hodgkin lymphoma and glandular structures (H & E; original magnification 400×); b) immunostaining showing neoplastic cells positive for CD20 (original magnification 400×)**.

**Figure 2:**
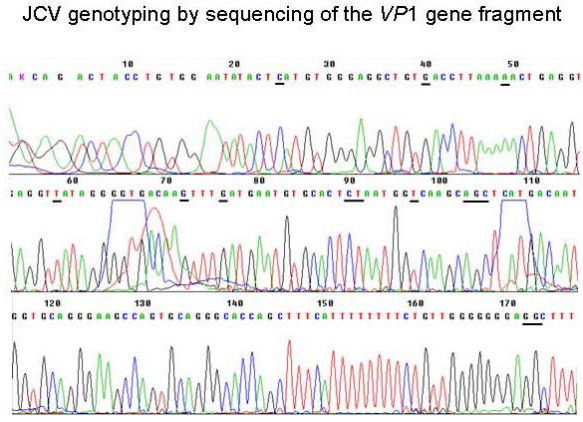
**Electropherogram of the JCV *VP1 *gene fragment sequence amplified from the primary adrenal lymphoma**. PCR amplification and sequencing was performed by using the JLP-15 and JLP-16 primers reported by Agostini *et al*. [10]. Nucleotides that are critical for genotyping are underlined.

The patient was a 77-yr woman with bilateral adrenal masses which were incidentally discovered at abdominal ultrasonography performed for dispepsia and weigth loss. The past history of the patients was unremarkable except hypertension. Computed tomography (CT)-scan confirmed the presence of bilateral (right adrenal mass maximum diameter, 5 cm; left adrenal mass maximum diameter, 7 cm) solid and heterogeneous adrenal masses which probably infiltrated the liver. Brain and bone CT-scans were negative. Laboratory evaluation demonstrated the presence of anemia, increased erythrosedimentation rate, and the presence of monoclonal G immunoglobulins, while autoantibodies testing was negative. Endocrine evaluation demonstrated primary adrenal insufficiency, since all adrenal steroids were low and adrenocorticotropin levels were markedly elevated. Replacement therapy with cortisone was started, resulting in immediate improvement of symptoms. Repeated chest-abdominal CT-scan performed after 3 months showed increased mass size with areas of colliquation and the presence of left pleural effusion. Bilateral nonvisualization at adrenal scintiscan suggested the presence of malignant or space-occupying adrenal lesions [8]. Fine needle aspiration biopsy was not performed because bilateral adrenocortical carcinoma or metastases were suspected based on CT-scan and scintigraphic appearance. At 5 months from diagnosis, the patient died because of advanced disease. Postmortem examination revealed bilateral involvement of adrenal glands by encapsulated masses with a solid, friable, grayish-white cut surface and extensive necrosis. Microscopic features consisted of large transformed lymphoid cells with pleomorphic vesicular nuclei with prominent nucleoli (Fig. 1a). Upon immunohistochemical analysis, the neoplastic cells expressed the B cell marker CD20 (Fig. 1b). A diagnosis of diffuse large B-cell non-Hodgkin lymphoma was made. To investigate whether oncogenic viruses were involved in the disease, both left and right adrenal masses were examined for the presence of all human herpesviruses and polyomaviruses by quantitative real-time PCR, as reported [7]. EBV DNA was present at high titre (about 100 genome copies/cell) and JCV DNA at lower titre (about 0.1 genome copy/cell) in both adrenal masses. EBV genotyping by PCR amplification of the *EBNA-2 *and *EBNA-3B *genes [9] demonstrated EBV type 1, while sequencing of the JCV *VP1 *gene [10] classified JCV as type 1B (Fig. 2), which are the genotypes most commonly found in our country. Moreover, PCR-amplification of both large T antigen (TAg) and the VP1 sequences suggested that the entire JCV genome was present. EBV and JCV DNA was not detected in other tissues (lymph nodes, spleen, liver, kidney). Both immunohistochemical staining and western blot analysis of JCV TAg expression in lymphoma samples, which were performed with an anti-SV40 TAg cross-reacting antibody (Calbiochem, Anti-SV40 T Antigen Ab-2, Pab416) as reported [7], gave negative results. It cannot however be excluded that lack of JCV TAg detection was due to the relatively low number of JCV-positive cells and to the poor quality of tissue samples.

While EBV is a recognized causative agent of B-cell lymphomas, the association between JCV and hematologic malignancies is matter of debate. A potential implication of JCV in B-cell lymphomas is suggested by its oncogenicity *in vitro *and in animal models [11] and by its ability to infect B-lymphocytes [12], where it seems to cause chromosomal damage [13]. Indeed, JCV DNA and gene expression have been detected in 4 of 63 (6%) non-Hodgkin lymphomas and in 1 of 20 (5%) Hodgkin lymphomas [14], in a primary B-cell lymphoma of the central nervous system occurring in a patient with AIDS and concurrent progressive multifocal leucoencephalopathy [15], and in 22 of 27 (81%) primary B-cell lymphomas of the central nervous system, including 14 which also contained EBV DNA [16]. However, it remains unknown whether JCV has a causal role in human neoplasia. Our report of a primary adrenal B-cell lymphoma positive for both JCV and EBV DNA, even if it does not provide evidence for a causal relationship between viral infection and development of lymphoma, could support the hypothesis by Del Valle *et al*. [16] that JCV might act as a co-factor of EBV in B-cell transformation. However, since we did not evaluate whether the patient had systemic JCV infection before death, we cannot exclude a secondary involvement of lymphoma cells in the course of a systemic infection due to JCV reactivation in a terminally-ill patient. This is also suggested by the relatively low JCV DNA load (0.1 copies/cell) in tumor tissues.

In conclusion, we demonstrated the presence of both EBV and JCV genome sequences in a diffuse large B-cell non-Hodgkin lymphoma of the adrenal gland. Further studies in other cases are encouraged to clarify the relevance of EBV and JCV coinfection in the pathogenesis of this rare form of extranodal B-cell lymphoma.

## Competing interests

The authors declare that they have no competing interests.

## Authors' contributions

LB performed clinical investigation, coordinated the study and draft the manuscript; MT carried out molecular tests; FM carried out pathological examination; VG carried out immunohistochemical analyses; GP participated in the coordination of the study and contributed to the preparation of manuscript. All authors read and approved the final manuscript.

## References

[B1] Wang J, Sun NC, Renslo R, Chuang CC, Tabbarah HJ, Barajas L, French SW (1998). Clinically silent primary adrenal lymphoma: A case report and review of the literature. Am J Hematol.

[B2] Mantzios G, Tsirigotis P, Veliou F, Boutsikakis I, Petraki L, Kolovos J, Papageorgiou S, Robos Y (2004). Primary adrenal lymphoma presenting as Addison's disease: case report and review of the literature. Ann Hematol.

[B3] Ozimek A, Diebold J, Linke R, Heyn J, Hallfeldt K, Mussack T (2008). Bilateral primary adrenal non-Hodgkin's lymphoma and primary adrenocortical carcinoma – Review of the literature. Preoperative differentiation of adrenal tumors. Endocr J.

[B4] Singh D, Kumar L, Sharma A, Vijayaraghavan M, Thulkar S, Tandon N (2004). Adrenal involvement in non-Hodgkin's lymphoma: four cases and review of literature. Leuk Lymphoma.

[B5] Schreiber CS, Sakon JR, Simião FP, Tomarchio MP, Huayllas M, Pereira LC, Stella LC, Santomauro AC, Bueno SS, Fraige FF (2008). Primary adrenal lymphoma: a case series study. Ann Hematol.

[B6] Ohsawa M, Tomita Y, Hashimoto M, Yasunaga Y, Kanno H, Aozasa K (1996). Malignant lymphoma of the adrenal gland: its possible correlation with the Epstein-Barr virus. Mod Pathol.

[B7] Barzon L, Trevisan M, Masi G, Pacenti M, Sinigaglia A, Macchi V, Porzionato A, De Caro R, Favia G, Iacobone M, Palù G (2008). Detection of polyomaviruses and herpesviruses in human adrenal tumors. Oncogene.

[B8] Barzon L, Scaroni C, Sonino N, Fallo F, Gregianin M, Macrì C, Boscaro M (1998). Incidentally discovered adrenal tumors: endocrine and scintigraphic correlates. J Clin Endocrinol Metab.

[B9] Lee HS, Chang MS, Yang H-K, Lee BL, Kim WH (2004). Epstein-Barr virus-positive gastric carcinoma has a distinct protein expression profile in comparison with Epstein-Barr virus-negative carcinoma. Clin Cancer Res.

[B10] Agostini HT, Deckhut A, Jobes DV, Girones R, Schlunck G, Prost MG, Frias C, Pérez-Trallero E, Ryschkewitsch CF, Stoner GL (2001). Genotypes of JC virus in East, Central and Southwest Europe. J Gen Virol.

[B11] White MK, Khalili K (2005). Expression of JC virus regulatory proteins in human cancer: potential mechanisms for tumourigenesis. Eur J Cancer.

[B12] Monaco MC, Atwood WJ, Gravell M, Tornatore CS, Major EO (1996). JC virus infection of hematopoietic progenitor cells, primary B lymphocytes, and tonsillar stromal cells: implications for viral latency. J Virol.

[B13] Neel JV, Major EO, Awa AA, Glover T, Burgess A, Traub R, Curfman B, Satoh C (1996). Hypothesis: "Rogue cell"-type chromosomal damage in lymphocytes is associated with infection with the JC human polyoma virus and has implications for oncogenesis. Proc Natl Acad Sci USA.

[B14] Hernández-Losa J, Fedele CG, Pozo F, Tenorio A, Fernández V, Castellví J, Parada C, Ramón y Cajal S (2005). Lack of association of polyomavirus and herpesvirus types 6 and 7 in human lymphomas. Cancer.

[B15] Gallia GL, DelValle L, Laine C, Curtis M, Khalili K (2001). Concomitant progressive multifocal leucoencephalopathy and primary central nervous system lymphoma expressing JC virus oncogenic protein, large T antigen. Mol Pathol.

[B16] Del Valle L, Enam S, Lara C, Miklossy J, Khalili K, Gordon J (2004). Primary central nervous system lymphoma expressing the human neurotropic polyomavirus, JC virus, genome. J Virol.

